# Functional impairment associated with self-stigma in severe mental illness: a cross-sectional study in Tunisia

**DOI:** 10.1192/j.eurpsy.2026.11709

**Published:** 2026-07-31

**Authors:** Y. Rebei, M. Touzri, S. Ben Othman, H. Mami, A. Aissa

**Affiliations:** 1 Psychiatry, Mohamed Taher Maamouri Hosiptal, Nabeul; 2 Faculty of Medicine of Tunis, Tunis, Tunisia

## Abstract

**Introduction:**

Self-stigma is a major psychosocial barrier in the recovery of individuals with severe mental illness. By internalizing public stereotypes, patients may experience reduced self-efficacy, social withdrawal, and impaired daily functioning. However, data on this association remain scarce in North African contexts.

**Objectives:**

To assess the association between self-stigma and functional impairment among patients with severe mental illness (SMI) in a Tunisian clinical setting. among patients with severe mental illness in a Tunisian clinical setting.

**Methods:**

A descriptive cross-sectional study was conducted from June to December 2023 in the Psychiatry Department A of Razi Hospital (Tunisia). We recruited clinically stable patients meeting DSM-5 criteria for SMI (schizophrenia, bipolar disorder, or major depressive disorder) were included. Self-stigma was assessed using the *Internalized Stigma of Mental Illness (ISMI)* scale, functioning with the *Functioning Assessment Short Test (FAST).* Data were analyzed with SPSS-26 using Pearson’s correlations and multiple linear regression.

**Results:**

Seventy patients were included. The mean age of participants was 46.2 ± 13 years with a gender ratio of 1.3. Moderate to severe stigma was found in 57% of patients, with a mean score of 2.6 ± 0.5. The average total functioning score was 32.8 ± 17.8, indicating overall function impairment in the majority of patients (80%). Higher self-stigma was significantly associated with poorer global functioning (p < 0.05), particularly in professional and cognitive domains.

**Conclusions:**

The survey showed a notable prevalence of internalized stigma among patient with severe MI. Self-stigma applies a significant influence in overall functioning in patients with severe mental illness.

**Disclosure of Interest:**

None Declared

